# Phylogenetic, Molecular, and Functional Characterization of PpyCBF Proteins in Asian Pears (*Pyrus pyrifolia*)

**DOI:** 10.3390/ijms20092074

**Published:** 2019-04-26

**Authors:** Mudassar Ahmad, Jianzhao Li, Qinsong Yang, Wajeeha Jamil, Yuanwen Teng, Songling Bai

**Affiliations:** 1 Department of Horticulture, Zhejiang University, Hangzhou 310058, Zhejiang, China; ahmad_mudassar@zju.edu.cn (M.A.); 21316043@zju.edu.cn (J.L.); qsyang@zju.edu.cn (Q.Y.); 11616122@zju.edu.cn (W.J.); 2 The Key Laboratory of Horticultural Plant Growth, Development and Quality Improvement, the Ministry of Agriculture of China, Hangzhou 310058, Zhejiang, China; 3 Zhejiang Provincial Key Laboratory of Integrative Biology of Horticultural Plants, Hangzhou 310058, Zhejiang, China

**Keywords:** asian pears, CBF, gene functions, CRT/DRE binding sites

## Abstract

C-repeat binding factor/dehydration-responsive element (CBF/DRE) transcription factors (TFs) participate in a variety of adaptive mechanisms, and are involved in molecular signaling and abiotic stress tolerance in plants. In pear (*Pyrus pyrifolia*) and other rosaceous crops, the independent evolution of CBF subfamily members requires investigation to understand the possible divergent functions of these proteins. In this study, phylogenetic analysis divided six *PpyCBFs* from the Asian pear genome into three clades/subtypes, and collinearity and phylogenetic analyses suggested that *PpyCBF3* was the mother CBF. All *PpyCBFs* were found to be highly expressed in response to low temperature, salt, drought, and abscisic acid (ABA) as well as bud endodormancy, similar to *PpyCORs* (*PpyCOR47*, *PpyCOR15A*, *PpyRD29A*, and *PpyKIN*). Transcript levels of clade II *PpyCBFs* during low temperature and ABA treatments were higher than those of clades I and III. Ectopic expression of *PpyCBF2* and *PpyCBF3* in *Arabidopsis* enhanced its tolerance against abiotic stresses, especially to low temperature in the first case and salt and drought stresses in the latter, and resulted in lower reactive oxygen species (ROS) and antioxidant gene activities compared with the wild type. The increased expression of endogenous ABA-dependent and -independent genes during normal conditions in *PpyCBF2*- and *PpyCBF3*-overexpressing *Arabidopsis* lines suggested that *PpyCBFs* were involved in both ABA-dependent and -independent pathways. All *PpyCBFs*, especially the mother CBF, had high transactivation activities with 6XCCGAC binding elements. Luciferase and Y1H assays revealed the existence of phylogenetically and promoter-dependent conserved *CBF–COR* cascades in the pear. The presence of a previously identified CCGA binding site, combined with the results of mutagenesis of the CGACA binding site of the *PpyCOR15A* promoter, indicated that CGA was a core binding element of *PpyCBFs*. In conclusion, PpyCBF TFs might operate redundantly via both ABA-dependent and -independent pathways, and are strongly linked to abiotic stress signaling and responses in the Asian pear.

## 1. Introduction

C-repeat binding factors/dehydration-responsive elements (CBFs/DREs) constitute a subfamily of the Apetala1/ethylene responsive factor (AP1/ERF) family and are characterized by the presence of one AP2 domain [1] that contains 60–70 highly conserved amino acid residues [2]. All CBFs have CBF signature motifs (PKK/RPAGRxKFxETRHP and DSAWR) that distinguish these factors from other AP1/ERF members harboring an AP2 domain [3]. This CBF motif specifically binds to the dehydration-responsive/C repeat (DRE/CRT) element (CCGAC) of downstream genes to regulate their expressions [4]. CBFs have a well-known role in cold response and acclimation in both herbaceous [5] and woody [6] plants. Studies on the poplar (*Populus trichocarpa),* eucalyptus (*Eucalyptus globulus*), grape *(Vitis vinifera),* sweet cherry *(Prunus avium)*, birch (*Betula pendula*), citrus (*Citrus paradisi*), and dwarf apple *(Malus baccata)*, have revealed that the cold acclimation function of CBF is highly conserved in these woody plants [7,8]. Nevertheless, several recent studies have suggested that the multiple CBF paralogs that have evolved in plants might perform different functions [9]. In this aspect, (i) CBF paralogs can influence each other’s expressions. In *Arabidopsis*, for example, *AtCBF2* negatively regulates the expressions of *AtCBF1* and *AtCBF3* [10]. (ii) In addition, CBF paralogs have different tissue specificities and expression times following cold stress. For example, *PtCBF2* and *PtCBF4* in poplars were detected only in leaves, whereas *PtCBF1* and *PtCBF3* were also expressed in leaves, stems, and dormant buds [11]. A similar result has also been reported in grapes, where *Vitis CBF4* was present in mature leaves and buds, while *Vitis CBF1*, *CBF2*, and *CBF3* were only found in young leaves and buds [12,13]. (iii) Several *CBF* genes have also been found to be induced by other abiotic stresses (drought and salt) and molecular signals (such as abscisic acid signaling). These include *GmDREB1G-1* and *GmDREB1G-2* in soybeans [14], *VrCBF1* and *VrCBF4* in grapse [9], *MbDREB1A* in dwarf apples [15], and *AtDDF1*, *AtDDF2* [16], and *AtCBF4* [17] in *Arabidopsis*. (iv) Overexpressed CBF paralogs from other species conferred various levels of abiotic stress tolerance on plants. For example, overexpression of both *VrCBF1* and *VrCBF4* enhances abiotic stress tolerance in *Arabidopsis*, but *VrCBF1* is mainly responsible for drought tolerance, while *VrCBF4* confers most of the cold tolerance [9].

A core set of robustly stress-responsive plant genes, known as *COR* (cold-regulated), *RD* (responsive to dehydration), and *KIN* (cold-induced), have been identified from numerous differential screening and cloning studies over the years. Many *COR* genes contain one or more similar CRT (CCGAC) elements in their promoters, which are also found in *CRT/DRE* genes, and interestingly, they all have abiotic stress responsiveness [18]. Abiotic stress rapidly induces CBFs, which then activate various downstream cold-responsive (*COR*) genes whose products collectively increase a plant’s abiotic tolerance capacity through necessary physiological and biochemical alterations [19]. The cold-stress induction of *CBF* and *COR* genes is also regulated by the circadian clock [20]. An important feature of abiotic stresses, especially low temperature, is a hyperosmotic signal that causes the phytohormone abscisic acid (ABA) to accumulate. ABA in turn provokes many adaptive responses, such as bud endodormancy, in plants [21]. Low temperatures and ABA have recently been reported to synergistically promote cold-hardiness and CBF expression in dormant grape buds [21]. These adaptive mechanisms are not only affected by ABA contents, but also by ABA signaling pathways [22]. For example, high ABA levels lead to endodormancy [23], inhibition of ABA pathways promotes germination and lateral root formation [24], while the reduction of ABA enhances water transpiration through stomatal pores [25].

Adaptive mechanisms, molecular signaling, and tolerance to abiotic stresses are also determined by many up- and downstream transcription factors of *CBF* genes. During the adaptive process of bud endodormancy in pears, for example, *PpICE3* works upstream of *PpCBF1*, while *PpCBF1, PpCBF2* and *PpCBF4* activate downstream *PpDAM1* and *PpDAM3* genes that induce endodormancy by inhibiting *PpFT2.* Meanwhile, microRNA *miR6390* degrades dormancy associated MADS (DAM) box genes to release endodormancy [22,26]*. MdMYB* and *MdHY5* in apples and *PbeNAC1* in pear have also been found to be involved in the regulation of *CBF* genes and the acquisition of abiotic stress tolerance [27,28,29]. In regards to molecular signals such as ABA, the PYR/RCAR–PP2C complex [30] inhibits PP2C [31] and activates SnRK2s, which not only target ABA-responsive genes (*ABF*/*ABI5*-type basic/region leucine zipper) [32], but also phosphorylate *ICE1* to activate CBF–COR cascades and promote plant tolerance through ABA signaling [33]. During abiotic stress, many transcription factors, i.e., COLD1, NAC, bHLH, ICE1, MYB, SnrK2, ABF, HOS1, and SIZ1, have been found to function upstream of CBFs, while ADF, ZAT, LOS, SFR, and RAP function downstream to induce plant tolerance [34]. Consequently, CBF is the central regulator of plant adaptation and abiotic stress tolerance via both ABA-dependent and -independent pathways [15].

*Pyrus* germplasm resources, which are distributed worldwide, are most plentiful in China, especially in the western and southwestern mountainous areas [35,36]. Numerous genes and TFs with functions related to plant dispersal, adaptation to natural habitats, and stress tolerance had been identified and characterized in plants, including AREB/ABF, MYB, AP2/EREBP, bZIP, HSF, CBF/DREB, MYC, HB, NAC, and WRKY. Among them, the CBF/DREB subfamily occupies a major position in both herbaceous [5] and woody [6] plants. The complete CBF subfamily and the possible divergent functions of its members have never been fully studied in rosaceous groups. In this study, we identified 15 *PpyCBFs* from the pear genome database, but were unable to predict their functions through phylogenetic analysis. Hence, we tested the hypothesis to know whether all *PpyCBF* paralogs had different functions or not. We therefore selected six of the 15 *PpyCBFs* after characterization and checked their responses to abiotic stresses, ABA treatment, and bud endodormancy compared with abiotic stress-responsive *PpyCOR* genes. We also generated *PpyCBF2-* and *PpyCBF3*-overexpressing *Arabidopsis* plants and analyzed their abiotic stress tolerances, endogenous gene expressions, and ROS accumulations. After checking the binding activity of all *PpyCBFs* with the *cis*-element (CCGAC), we also studied their possible abiotic regulatory pathways and binding sites in pears.

## 2. Results

### 2.1. Identifications and Characterizations of PpyCBF Subfamily

**Figure 1:**
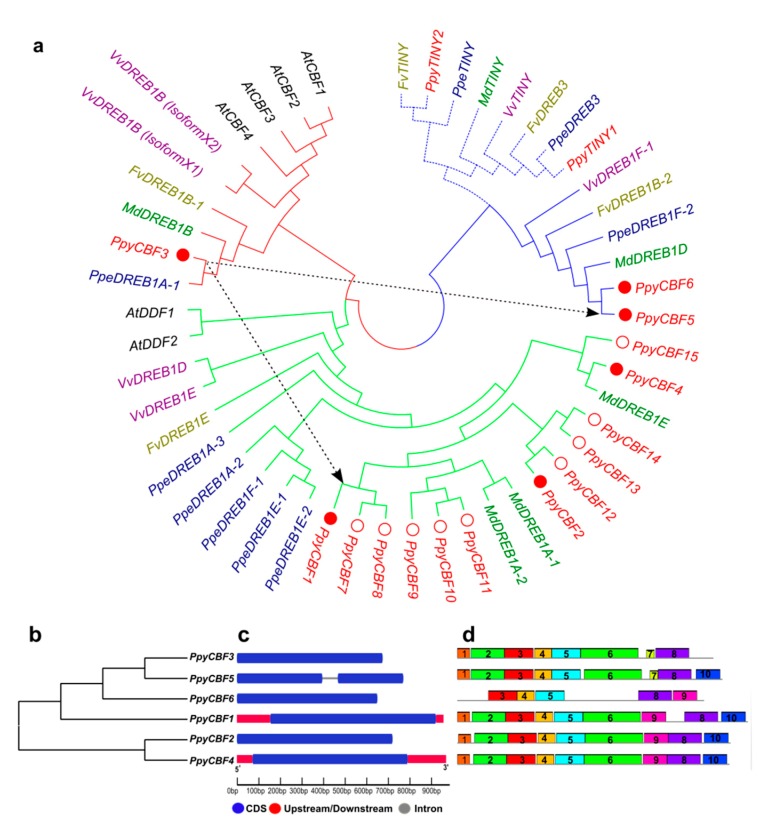
Identification and characterization of *PpyCBFs*. (**a**) Phylogenetic analysis of *PpyCBF* transcription factors with similar TFs of *Arabidopsis* (*At*), *Malus* (*Md*), *Prunus* (*Ppe*), *Fragaria* (*Fv*), and *Vitis* (*Vv*) species. Red, green, and blue colors indicate clades/subtypes I, II, and III of CBFs, respectively, while compact and hollow red circles indicate selected and rejected *PpyCBFs*, respectively. Arrow lines indicate the evolution of clades II and III from clade I. (**b**) Phylogenetic analysis of selected *PpyCBFs*. (**c**) Gene structure of *PpyCBFs*. Blue, black, and red lines indicate exon, intron, and upstream/downstream sections in gene structure. (**d**) Protein motif: Schematic diagrams of possible conserved motifs (1–10) in *PpyCBF* proteins, indicated by different colors.

To identify *PpyCBFs*, we first carried out a hidden Markov model search against the pear genome database. This approach identified 15 PpyCBF TFs, which were then subjected to phylogenetic analysis and further confirmation of their sequence identities and chromosomal positions. Pairwise sequence identities among isolated *PpyCBFs* were all very high, ranging from 0.271 (*PpyCBF9* and *PpyCBF10* vs. *PpyCBF12*) to 0.994 *(PpyCBF15* vs. *PpyCBF4*) (Table S1). Sequences that had an identity >0.90 and were on the same phylogenetic branch (*PpyCBFs 7,8,9,10,11,12,13,14*), incomplete (*PpyCBF12*), or on a scaffold (*PpyCBFs 7,8,10,11,13,14,15*) were eliminated from further analysis, whereas their corresponding sequences, i.e., *PpyCBFs 1–6*, were retained (Figure 1a, Table S1). To explore evolutionary relationships within the isolated subfamily, we first constructed a phylogenetic tree of sequences of similar candidates in *Pyrus* (*Ppy*), *Arabidopsis* (*At*), *Malus* (*Md*), *Prunus* (*Ppe*), *Fragaria* (*Fv*), and *Vitis* (*Vv*). The phylogenetic analysis distributed the *PpyCBFs* into three main clades/subtypes: *PpyCBF3* in clade I, *PpyCBFs 1*,*2*,*4* in clade II, and *PpyCBF5* and *PpyCBF6* in clade III. Interestingly, *PpyCBFs*, along with *CBFs* of other rosaceous crop species, appeared to be evolved independently of model crop *CBFs* (*AtCBFs 1*–*4*). With the exception of *PpyCBF3*, which was clustered in clade I with *Arabidopsis CBFs*, all other *PpyCBFs* were placed in clades II and III with *MdDREBs* and *PpeDREBs* (Figure 1a). This independent evolution of *PpyCBFs* suggested their potential divergent functions and served as the impetus for our study to explore and elucidate the regulation of this family in pears.

Since *PpyCBFs* belong to the AP2/ERF family, we performed a collinearity analysis of the entire family to understand *PpyCBF* evolution and gene duplication (Figure S1a). We found 68 duplicated AP2/ERF pairs. Among them, two pairs, i.e., *Pbr013924*(*PpyCBF3*):*Pbr032764*(*PpyCBF5*) and *Pbr013924*(*PpyCBF3*):*Pbr021781*(*PpyCBF1*), belonged to its *PpyCBF* subfamily (Figure S1b). These results suggest that clades II and III of CBFs, i.e., *PpyCBF1* and *PpyCBF5*, evolved from *PpyCBF3*, which was found in an ancestral clade with both monocot and dicot plants (Figure 1a). To examine diversification in gene structures and uncover potential conserved motifs in these selected *PpyCBFs*, we constructed another phylogenetic tree, which revealed that both duplicated *PpyCBF3* and *PpyCBF5,* and *PpyCBF2* and *PpyCBF4* had potentially similar functions. In addition, *PpyCBF5* together with *PpyCBF6* were in a sister relationship with a cluster comprising *PpyCBF1* and *PpyCBFs 2*,*4*, with the branch leading to these genes in turn joined to the ancestral *CBF* (Figure 1b). Regarding gene structures and conserved motifs, *PpyCBF5* was the only gene with just one intron. All the others had exonic regions (Figure 1c). Alignment of *PpyCBFs* in each phylogenetic clade revealed 10 different types of common motifs (Figure 1d). These findings indicate that *PpyCBFs* in the same clade have similar gene structures and motifs, and possibly similar functions.

### 2.2. Strong Induction of PpyCBF Transcription by Various Abiotic Stresses and ABA Treatment

**Figure 2:**
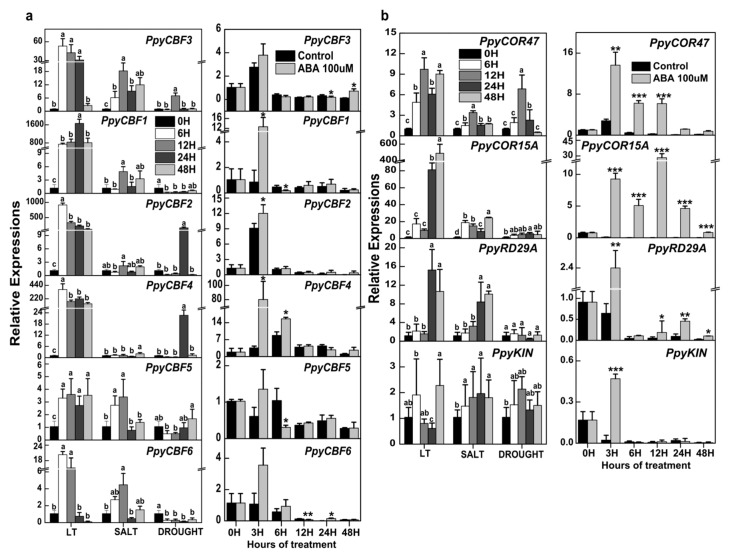
Relative expressions of *PpyCBFs* and *PpyCORs* during abiotic stresses and exogenous abscisic acid (ABA). (**a**) Expression analysis of *PpyCBFs* during abiotic stresses (cold, salt, and drought) and ABA according to their phylogenetic clades. (**b**) Expression analysis of *PpyCOR47, 15A*, *RD29A*, and *KIN* in the same samples for comparison study. Both relative expressions were normalized to *PpyActin* expression level. Error bars indicate standard errors from three biological replicates (* *p* < 0.05, ** *p* < 0.01, *** *p* < 0.001) while means with different letters had significant differences (*p* < 0.05).

To better understand the functions of *PpyCBFs*, we examined transcript levels of *PpyCBFs* in explants of *Pyrus pyrifolia* ‘Dangshan Suli’ subjected to different abiotic stress treatments, i.e., low temperature (4 °C), drought (15% polyethylene glycol (PEG)) and salt (200 mM NaCl), for 0, 6, 12, 24, and 48 h. qRT-PCR analysis revealed that the expressions of all six *PpyCBF* genes were induced by all abiotic stresses, but each gene responded differently to various stresses depending on its associated clade (Figure 2a). During cold treatment, expressions of *PpyCBFs* were all constant from 6 to 48 h and significantly higher than the control, with relative abundances of clade II CBFs which were much higher (~200–1600) than those of clade I and II CBFs (~2–50). During salt treatment, all *PpyCBFs* were statistically at their maximums after 12 and 48 h except for *PpyCBF4* (which peaked only at 48 h). The responses of clade I and III *PpyCBFs* were higher at early stages of salt stress than those of clade II *PpyCBFs*. Under drought conditions, *PpyCBF3* (12 h)*, PpyCBF2* (24 h)*, PpyCBF4* (24 h), and *PpyCBF5* (48 h) were accentuated, while *PpyCBF1* and *PpyCBF6* were downregulated. To determine whether *PpyCBFs* respond to ABA, we also tested their expressions in pear calli after 0, 3, 6, 12, and 48 h of ABA treatment (100 µM). Notably, all *PpyCBFs* had responses to ABA after 3 and 48 h. Short-term ABA exposure significantly promoted the expressions of clade II *PpyCBFs*, whereas longer exposure significantly induced the members of the other two clades *(PpyCBF3* and *PpyCBF6)*. Expression levels of clade II *PpyCBFs* were much higher than those of clades I and III. Significant downregulation of *PpyCBF3* (24 h), *PpyCBF1* (24 h), *PpyCBF5* (6 h), and *PpyCBF6* (12 h) was also observed during ABA treatment of pear calli (Figure 2a). In summary, clade I and III *PpyCBFs* exhibited higher levels of transcripts during salinity and drought treatments, whereas clade II *PpyCBF* transcripts were more abundant during low temperature and ABA stresses.

We also compared the expressions of *PpyCBFs* with those of *COR* genes (*PpyCOR47, PpyCOR15A, PpyRD29A*, and *PpyKIN*) during ABA treatment and abiotic stress. qRT-PCR analysis uncovered highly significant expressions of *PpyCORs* during cold, salt, and drought stresses, the exception being *PpyRD29A* during drought. Likewise, *PpyCORs* exhibited a highly significant, constant response throughout ABA treatment (Figure 2b). To confirm the above results and check the stress status of explants and calli, we measured expression levels of antioxidant genes (*PpySOD, PpyPOD, PpyAPX*, and *PpyCAT*) during abiotic stress and those of ABA-responsive genes (*PpyCYP707A-2, PpySnRK2-1* and *PpySnRK2-4, PpyABi5*, and *PpyPYL-2*) subjected to ABA treatment (Figure S2). The expressions of all these genes were found to be high. These results not only verify the effectiveness of the treatments, but also suggested that all *PpyCBFs* were differentially induced according to their clades during abiotic stresses and ABA treatments.

To understand the possible transcriptional regulatory cascades of *PpyCBFs*, we also analyzed their promoters. We detected numerous *cis* elements responsive to biotic and abiotic stresses, molecular signaling, and plant adaptation in promoters of *PpyCBF* transcription factors related to cold, salt, drought, oxidation, light, heavy metals, pathogens, heat, ABA, giberllic acid, and auxin, namely, ABI3/VP1, AP2/EREBP, AP2/RAV, ARF, bHLH, bZIP, ERF, GATA, MADS, MYB, MYC, NAC, TCP/PCF1, and WRKY *cis* elements (Table 1 and Table S2). We found varying degrees of differences between the types and numbers of *PpyCBF* regulatory elements. The presence of these *cis* elements suggests that ABA and stress-inducible expressions of *PpyCBFs* are transcriptionally regulated.

### 2.3. Increased Transcripts of PpyCBFs Induced by Low Temperature and ABA during Pear Bud Endodormancy

**Figure 3:**
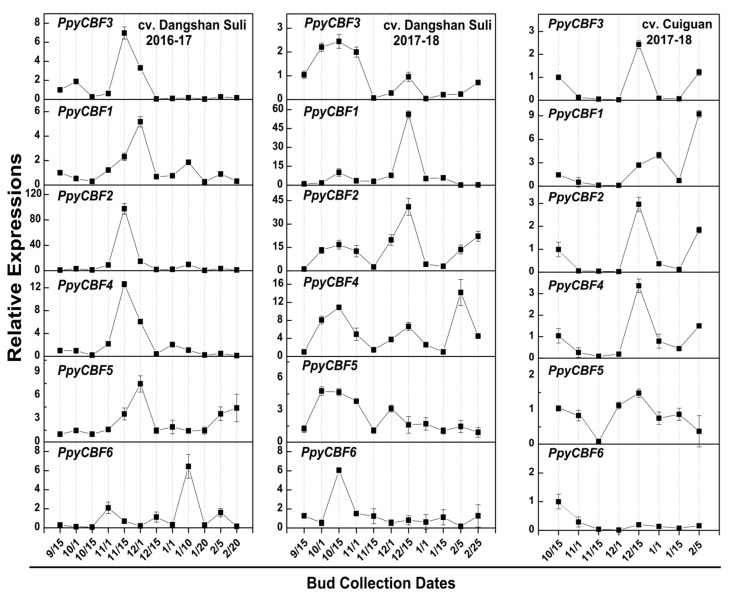
Relative expressions of *PpyCBFs* during bud endodormancy in *Pyrus pyrifolia* cv. ‘Dangshan Suli’ and ‘Cuiguan’ during two successive years 2016–2017 and 2017–2018. Buds were collected from September 15 to February 25 with about 15-day intervals. The data were normalized to *PpyActin* levels and the mean expression value was premeditated from four independent replicates. The standard deviation was shown by vertical bars.

As inferred from the above results, all *PpyCBFs* responded to ABA and low temperature, two basic factors for the establishment of bud endodormancy. We therefore also verified the expressions of *PpyCBFs* during the endodormancy period from September to February in Asian pear cultivars ‘Dangshan Suli’ and ‘Cuiguan’ at 15-day intervals in 2016–2017 and 2017–2018. During bud endodormancy, we observed two peaks in *PpyCBF* expression, the first one related to low temperature and the other dependent on ABA. In both pear cultivars, all *PpyCBFs* had their first expression peaks on January 1–12, 2017, and January 10–11, 2018, with their maximum expressions on November 15 and October 15 of the two respective years (Figure 3). As reported in our previous study [22], below-normal maximum and minimum temperatures were observed from October 15 to November 15 during 2016–2017, with the winter season also delayed in 2016–2017 compared with 2017–2018 (November vs. October). These events ultimately affected the transcription of *CBFs* during both years. Nevertheless, *PpyCBF* transcripts in both cultivars had their second expression peaks between January 1–20, 2017, and from December 1, 2017, to January 1, 2018, with maximums observed in the middle of January and December in the two successive years. This indicated ABA-dependent responses of *PpyCBFs* during bud endodormancy (Figure 3) because, in our previous study of ABA-responsive genes, *PpyNCED1*, *PpyCYP707A*-*3* and *PpyCYP707A*-*4*, and *PpyLs 2*,*3,6,7*,*8* were at their peaks on January 1–20 during bud endodormancy [23]. Interestingly, the relative abundances of clade II *PpyCBFs* during low temperature and ABA peaks were higher than those of clades I and III during both years in both cultivars, consistent with our results discussed earlier (Figure 2a).

To further clarify low-temperature and ABA responses of *PpyCBFs* during bud endodormancy, we rechecked the responses of the studied *PpyCORs* during pear bud endodormancy to verify their high expressions during low temperature and ABA treatments (Figure 2b). Similar to the *PpyCBFs*, all *PpyCORs* (*PpyCOR47*, *15A*, *RD29A*, and *KIN)* had expression peaks from November 15, 2016, to December 1, 2016, and from October 1, 2017, to November 1, 2017, corresponding to a low-temperature response, and from January 1–10, 2017, and from December 12, 2017, to January 1, 2018, corresponding to an ABA response, in both cultivars, with the exception of *PpyKIN* during 2016–2017 (Figure S3). The relative abundance of *PpyCOR15A* during low temperature and ABA peaks was higher than that of other *CORs* during low-temperature and ABA treatments (Figure 2b). These results not only reveal the responses of *PpyCBFs* and *PpyCORs* during bud endodormancy but also demonstrate their obvious correlation to each other.

### 2.4. Overexpressions of PpyCBF2 and PpyCBF3 Positively Regulate Abiotic Stress Tolerances in Transgenic Arabidopsis

**Figure 4:**
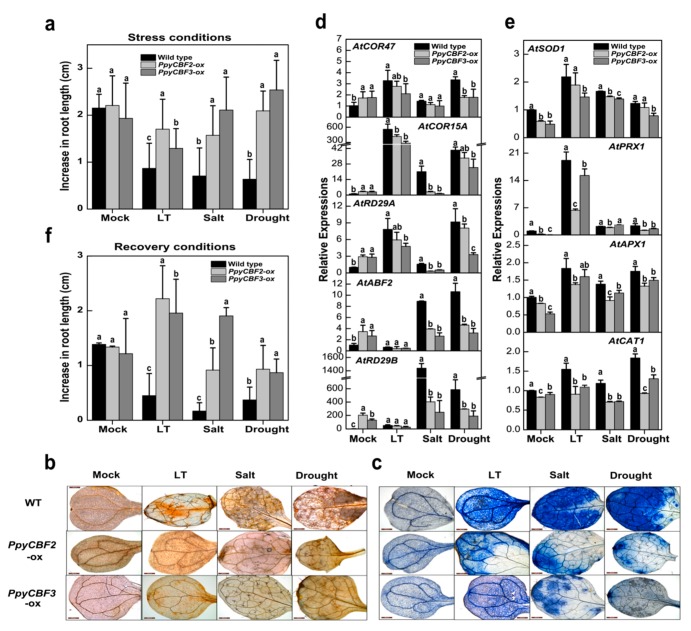
Overexpression analysis of *PpyCBFs 2* and *3* in *Arabidopsis* during abiotic stresses. (**a**) Increase in root length (cm) of wild type (WT) and overexpressed lines during low temperature (LT), salt, and drought treatments by using ImageJ software. Error bars indicate standard errors from three biological replicates. (**b**,**c**) Diaminobenzidine (DAB) and nitroblue tetrazolium (NBT) staining of WT and overexpressed leaves after abiotic stresses to check ROS accumulation where brown and blue spots indicate the presence of H_2_O_2_ and O2^•−^ in situ while the red bar scale represent 200 μm. (**d**,**e**) Endogenous gene expressions of ABA-independent (*AtCOR47, AtCOR15A* and *AtRD29A*), ABA-dependent (*AtABF2* and *AtRD29B*) and antioxidant genes (*AtSOD1, AtPRX1, AtAPX1* and *AtCAT1*) in WT and overexpressed lines during control and abiotic stresses, normalized to *AtPP2A* expression levels. (**f**) Increase in root length to monitor the recovery among overexpressed and WTs *Arabidopsis* under normal conditions after abiotic stresses. Error bars indicate standard error from three biological replicates. Means with different letters had significant differences (*p* < 0.05).

To test whether *PpyCBFs* overexpression positively enhances abiotic stress tolerance, pCAMBIA1301 overexpression constructs of *PpyCBF2* (the most transcriptionally activated CBF) and *PpyCBF3* (the mother CBF) were transformed into *Arabidopsis*. Consistent with abiotic stress assays, phenotypes of both *PpyCBF2-ox* and *PpyCBF3-ox* transgenic lines were superior in several respects to the wild type (Figure S4a). Ectopic expression of *PpyCBF2* and *PpyCBF3* led to highly significantly increased root lengths after treatment with low temperature (1.7 and 1.3 cm, respectively), salt (1.5 and 2.1 cm), and drought (2.0 and 2.5 cm) compared with wild-type plants (0.8, 0.7, and 0.6 cm under low temperature, salinity, and drought, respectively), whereas no differences were observed among wild-type, *PpyCBF2-ox*, and *PpyCBF3-ox* plants under non-stress conditions (2.1, 2.2, and 1.9 cm, respectively) (Figure 4a). Interestingly, *PpyCBF2-ox* plants under low temperature stress and *PpyCBF3-ox* plants under salinity and drought stress had more pronounced length increases relative to the wild type, but more growth retardation was observed in all plants during low temperature stress than during salt and drought stress.

To confirm the effect of *PpyCBF2-ox* and *PpyCBF3-ox* on endogenous *Arabidopsis* genes, we examined the expressions of three ABA-independent (*AtCOR47*/*RD17*, *AtCOR15a*, and *AtRD29A/COR78/LTI78*), two ABA-dependent (*AtABF2* and *AtRD29B*) and four antioxidant (*AtSOD1, AtPRX1, AtAPX1, AtCAT1*) genes. In *Arabidopsis* overexpressing either *PpyCBF2* or *PpyCBF3* under control or unstressed conditions, the ABA-dependent and -independent genes were significantly upregulated, and the antioxidant genes were downregulated (Figure 4d,e). Under each stress treatment, relative abundances of all stress-responsive and antioxidant genes were significantly lower in both overexpressing *Arabidopsis* lines, relative to the wild type (Figure 4e), while antioxidant gene expressions were higher in *PpyCBF3-ox* plants than in *PpyCBF2-ox* ones. To verify the above results, we investigated the accumulations of H_2_O_2_ and O_2_^•−^ by examining diaminobenzidine (DAB) and nitroblue tetrazolium (NBT) precipitation in *PpyCBF2-ox, PpyCBF3-ox*, and wild-type plants. Although no differences were apparent between wild-type and overexpressing plants under control conditions, more intense brown and blue precipitates were observed under abiotic stress in leaves of wild-type plants stained with DAB and NBT, respectively. 

The results of DAB and NBT staining indicate that overexpressing plants accumulated less H_2_O_2_ and O_2_^•−^ during abiotic stress than the wild type (Figure 4b,c). The more pronounced activity of major H_2_O_2_- and O2^•−^-scavenging enzymes (AtPRX, AtAPX, AtCAT and AtSOD) in wild-type plants was due to the higher accumulation of these toxic molecules, whereas the higher activity of antioxidant genes in *PpyCBF3-ox* plants indicated that scavenging of accumulated ROS was more successful in *PpyCBF3-ox* than in *PpyCBF2-ox* plants (Figure 4b,c,e).

After abiotic stress treatments, both wild-type and overexpressing plants were grown under control conditions for 7 days to monitor their recovery. Almost all CBF transgenic plants exhibited more pronounced prostrate growth during recovery than wild-type ones, which were found to be under severe stress (Figure S4b). After salt stress, both overexpressing lines experienced significant growth. Following low-temperature and drought treatments, *PpyCBF2-ox* and *PpyCBF3-ox* plants had significantly longer roots than their respective wild type (Figure 4f).

### 2.5. PpyCBF Transcriptional Activation of 6X C-Repeat Binding Sites and Stress-Responsive Genes

To examine *PpyCBF* abiotic regulatory cascades, we first measured the CRT-dependent transactivation activities of *PpyCBFs* in dual luciferase assays. For this analysis, full-length *PpyCBFs* were inserted into a SK vector, and 6X C-repeat binding sites (CCGAC) were inserted along with a 35S promoter into a LUC vector. We found that all *PpyCBFs* had transcriptional activities with the 6X C-repeat binding sites, with the ancestral CBF (*PpyCBF3*) showing the strongest interaction with these binding sites (Figure S5).

**Figure 5:**
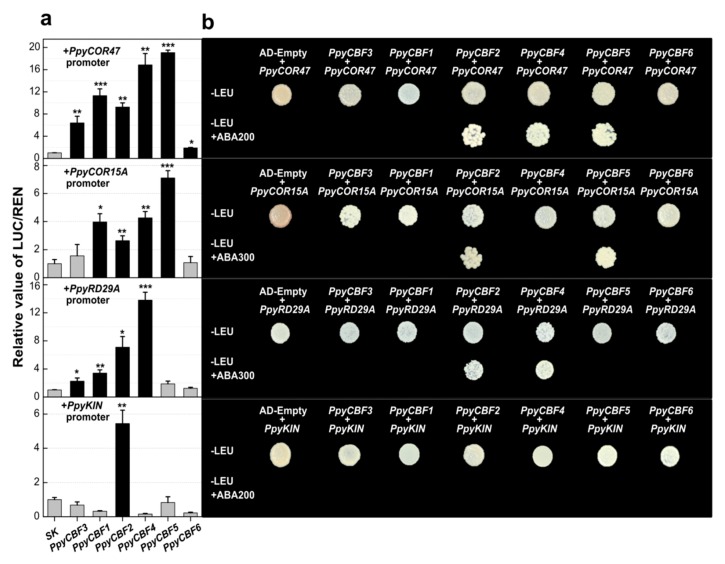
In vivo and in vitro regulations of *PpyCBFs* on the promoters of stress-related genes. (**a**) Dual luciferase assay to check the in vitro regulations. The ratio of firefly luciferase/renilla luciferase (LUC/REN) of the empty vector (pGreenII 0029 62-SK) plus promoter was used as calibrator (set as 1). Three independent experiments were done to verify the results. Error bars show SEs with at least four biological replicates, while asterisks show significant differences of genes SK with empty SK (* *p* < 0.05, ** *p* < 0.01, *** *p* < 0.001). (**b**) Y1H assay shows in vivo binding of *PpyCBFs* on *PpyCOR* promoters. Synthetic dropout (SD) medium without Leu and supplemented with 200 and 300 ng mL−1 ABA was used. Yeast grew on ABA-supplemented plates, indicating the possible direct interactions.

To further investigate possible transcriptional regulatory linkages involved in pear abiotic stress pathways, dual luciferase (in vitro) and Y1H (in vivo) assays were performed with *PpyCBF* and *PpyCOR* promoters. The dual luciferase assays revealed that *PpyCBFs 1*–*6*, *PpyCBFs 1,2,4*,*5*, *PpyCBFs 1*–*4*, and *PpyCBF2* could significantly transactivate the promoters of *PpyCOR47*, *PpyCOR15A*, *PpyRD29A*, and *PpyKIN*, respectively. Clade II *PpyCBFs* had high transcriptional activities with *PpyCOR47*, *15A*, and *RD29A*, while clade I and III *PpyCBFs* had little interaction with *PpyRD29A* (Figure 5a). In view of these results, Y1H assays were performed between *PpyCBF* genes and *PpyCOR* promoters. The Y1H results validated the direct interactions of *PpyCBFs 2,4*,*5* with *PpyCOR47*, *PpyCBFs 2* and *5* with *PpyCOR15A*, and *PpyCBFs 2* and *4* with *PpyRD29A* promoters, while no interactions were detected between *PpyKIN*–*PpyCBFs*. Interestingly, the ancestral CBF did not show any physical interaction with stress-responsive genes, while *PpyCBF2* was found to be the most active transcriptional regulator during abiotic stress signaling (Figure 5b).

### 2.6. PpyCBFs Can Also Bind at the TCGAC Binding Site in the PpyCOR15A Promoter

**Figure 6:**
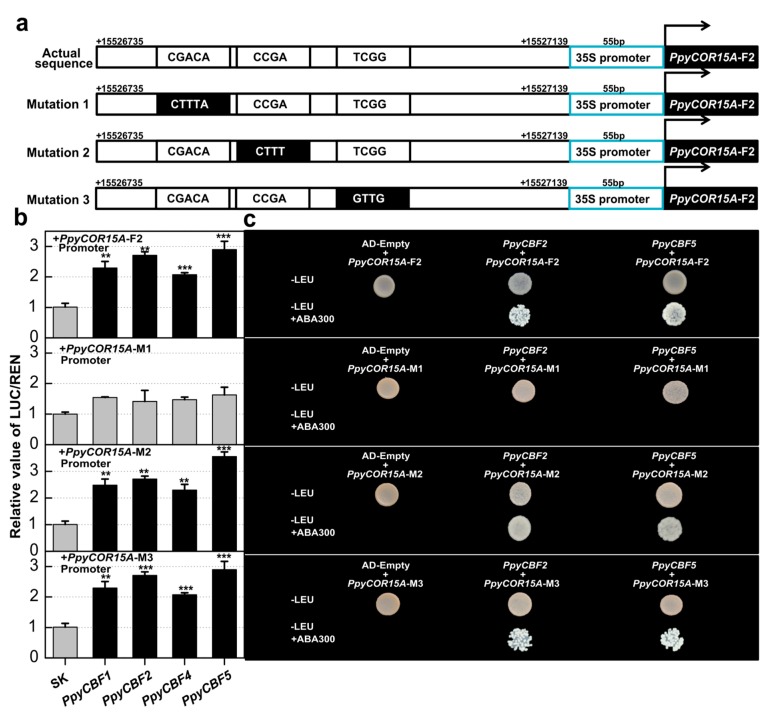
PpyCBFs can also bind at TCGAC binding site in the *PpyCOR15A* promoter. (**a**) Schematic diagrams of mutations at three different motif sites for *PpyCOR15A* promoters, indicated with mutation 1, 2, and 3. Possible CBF-binding sites in *PpyCOR15A* promoter are represented with white rectangles while mutations at these sites are represented by black rectangles. (**b**) Dual-luciferase assays were performed with actual and mutated promoters of the *PpyCOR15A* promoter. The ratio of LUC/REN of the empty vector (pGreenII 0029 62-SK) plus promoter was used as the calibrator (set as 1). Three independent experiments (with minimum four replicates) were performed to verify the results. Error bars show SEs with at least four biological replicates while asterisks show significant differences with empty SK (** *p* < 0.01, *** *p* < 0.001). (**c**) Y1H assay was performed to check physical interaction of PpyCBFs 2 and 5 with actual and mutated promoters of *PpyCOR15A*. Yeast grows on synthetic dropout without leucine but having Aureobasidin A 300 (SD/−leu + ABA300) indicating the possible direct interactions.

The above findings indicate that *PpyCBFs* have transcriptional activities with 6X CCGAC binding sites. According to an analysis of *PpyCOR* promoters, however, *PpyCOR15A* had no CRT binding site in its promoter region, but had high transcriptional activities with *PpyCBFs* (Table S3). To identify the unique *PpyCBF* binding site in the *PpyCOR15A* promoter, we therefore first divided the *PpyCOR15A* promoter into four fragments. We observed both in vitro and in vivo interactions of *PpyCBFs* with fragment 2 of *PpyCOR15A* (Figure 6b,c). We identified three possible CBF-binding sites in this region, CGACA, CCGA and TCCG, and mutated them into CTTTA, CTTT and GTTG, respectively (Figure 6a). Luciferase and Y1H assays proved that the mutation at the CGACA binding site reduced the transcriptional activities and physical interactions of all *PpyCBFs* with the *PpyCOR15A* promoter present at −615 to −610 bp from the start codon. No effects on transcriptional regulation or direct interactions were observed at the second and third mutation sites. Hence, *PpyCBFs* can also bind to the TCGAC binding site, and the deletion of one cytosine from the CRT binding site did not influence its binding activity with the *PpyCOR15A* promoter in pears.

## 3. Discussion

In this study, we isolated 15 PpyCBF TFs from the pear genome. On the basis of sequence identity, phylogeny, conserved domain sequence (CDS) completeness, and scaffold position, however, only six *PpyCBFs* genes were selected for further study (Figure 1 and Table S1). Several CBF-specific domains, especially AP2, had strong conservations in plants, ultimately reflecting their high levels of identity [1,4]. This result explains why many identical amino acid residues and homologous groups were also found among CBFs of pears (Table S1) and other crop species, such as *Arabidopsis*, soybeans, apples, grapes, and different grasses [9,10,14,37]. Phylogenetic analysis provided evidence of independent evolution and three main PpyCBF clades/subtypes, while collinearity analyses uncovered two duplicated gene pairs (Figure 1 and Figure S1). The first clade not only contained CBFs from dicot and monocot crop species, but also the collinear gene *PpyCBF3*. The presence of *PpyCBF3* in this first clade along with genes from both monocots and dicots, and the evolutionary relationship of this clade to the other two CBF clades suggested that *PpyCBF3* might be the ancestral CBF from which all other CBFs were derived during whole-genome duplication in pears prior to their divergence from apples. This result is similar to soybeans, where the presence of orthologs from both dicot and monocot plants suggests that *GmDREB1* clade/subtype 4 genes are the ancestral genes in the GmDREB1 family [14]. Rosaceous and *Arabidopsis* crop CBFs may have evolved completely independently of one another, as CBF regulation in woody plants appears to be more complex than that in herbaceous plants [11].

As mentioned above, *PpyCBFs* were found to have different predicted functions than those of *AtCBFs*, which was corroborated by abiotic stress and bud endodormancy experiments that revealed that *PpyCBFs 1*–*6* were not only induced by low temperature, salt, and drought stresses, but also by exogenous and endogenous ABA (Figure 2a and Figure 3). The predicted functions and expressions of these *PpyCBFs* were similar to those of *MbDREB1* in apples [15], *PaDREB1* in sweet cherries [38], *BrCBF* in non-heading Chinese cabbages [39], and *VviDREB1* in cowberries [40] during abiotic stress, but they were dissimilar to *AtCBFs 1*–*3* in *Arabidopsis*, which is only low-temperature responsive [10]. A proposed explanation for these expression changes is that cold, drought, and high salinity all cause osmotic stress [5]. In Japanese pears during bud endodormancy, we observed that the expressions of CBF/DREB4, DREB1E, DREB2, DREB2A, and DREB2D first peaked on December 24 and then suddenly declined on January 8, with a second expression peak on January 20 in both ‘TH3′ and ‘Hengshani’ cultivars [41]. We hypothesized that the first peak was low-temperature-responsive, while the second was ABA-responsive. To confirm in vivo functions of *PpyCBFs* in plants, we ectopically expressed two *PpyCBF* genes, *PpyCBF*2 and *PpyCBF*3, in *Arabidopsis.* We found that plants of the two exogenous *PpyCBF-ox Arabidopsis* lines had higher resistance to low temperature (10 °C), salt (50 mM), and drought (10%) stresses than the wild type (Figure 4a), similar to results in transgenic plants overexpressing DREB1s from apples, soybeans, grapes, and cabbages [9,14,15,39]. Interestingly, overexpression of *PpyCBFs* did not cause a dwarf phenotype in transgenic *Arabidopsis* grown on Murashige–Skoog (MS) medium (Figure S4), an outcome in agreement with observations from overexpression of *MbDREB1* genes in *Arabidopsis* [15]. One notable feature of low-temperature stress and CBF overexpression is that both cause marked growth retardation resulting from the promotion of GA catabolism by two CBF-regulated isoforms (*GA2ox3* and *GA2ox6*) and subsequent accumulation of DELLA proteins [42]. Some evidence suggests that at least a few CBF paralogs have evolved to execute different functions [9], which would explain the differential responses of *PpyCBF* paralogs to various stresses observed in our study (Figure 2a). In particular, *PpyCBFs* from clade II were not only more cold-responsive during abiotic stress and bud endodormancy, but they also exhibited higher resistance in overexpressing *Arabidopsis* to cold stress compared with salt and drought stresses. In contrast, clade I and III CBFs were highly salt- and drought-responsive and were more resistant in transgenic *Arabidopsis* to these stresses (Figure 2 and Figure 3). This situation is similar to soybeans, where the expressions of *GmDREB1* genes assigned to phylogenetic subtypes 1 and 2 were found to be induced by low-temperature, salinity, drought, and heat stresses, whereas those of subtype 4 were only induced by low temperature and salt [14].

The expression patterns of CBFs and CORs in pear are similar to those in other plant species [34]. Our qRT-PCR analysis revealed that *PpyCOR* expressions were increased not only by cold, salt, and drought stresses, but also by endogenous and exogenous ABA (Figure 2b). This result is unsurprising, as CBF-induced tolerance to cold, salt, drought, and ABA has been repeatedly correlated with increased expressions of *COR* genes [9]. Significantly higher amounts of *PpyCOR15a* and *PpyCOR47* transcripts were detected during abiotic stress, however, the reason why the expressions of *PpyRD29A* and *PpyKIN* did not follow the same trend as other *COR* genes is unclear. We note that specific information on all *COR* genes in pears are still limited. In regard to the effect of *PpyCBFs* on endogenous ABA-dependent and -independent genes, we observed significantly higher expressions of these genes under normal, unstressed conditions in *PpyCBF2-ox* and *PpyCBF3-ox* lines than in the wild type (Figure 4d). These findings suggest that *PpyCBF2* and *PpyCBF3* participate in both ABA-dependent and -independent pathways during abiotic stress signaling. Similar findings have also been reported for apples, grapes, and potatoes, where overexpressed *MbDREB1*, *VvCBF*, and *ScCBF1* significantly increase the expressions of ABA-independent (*AtCOR15a*, *AtRD29A*, *AtCOR6*.6, and *AtCOR47*) and ABA-dependent (*AtRD29B*, *AtRAB18*, *AtABI1*, and *AtABI2*) genes during normal conditions [9,15]. Interestingly, the expressions of all stress-responsive genes during abiotic stress conditions were significantly lower in overexpressing lines than the wild type, as the overexpressing lines had more resistance than the wild type because of the endogenous activation of *AtCOR* genes (Figure 4d).

Upon further investigation of transcriptional regulatory pathways of *PpyCBFs*, we uncovered their central role during abiotic stress signaling in pears (Figure 5 and Table 1). The results of our luciferase and Y1H assays indicated the existence of at least two main types of transcriptional interactions associated with CBF clades. In other words, all clade CBFs (except PpyCBF6) had interactions with *PpyCOR47* and *15A*, while clade II *PpyCBFs* had a stronger association with *PpyRD29A* compared with clades I and III. *PpyCBFs* were involved in the same CBF–COR cascades during abiotic stresses that are conserved in multiple plant species such as *Arabidopsis* and *Brachypodium*, with *AtCBF1*–*3* and *BdCBF1* showing interactions with *COR* genes by binding CRT/DRE (CCGAC) elements [34,37]. We also observed high transcriptional activities of all *PpyCBFs* with 6XCRT/DRE (CCGAC) binding sites. An analysis of *PpyCOR* gene promoters uncovered no CCGAC binding sites in the promoters of *PpyCOR15A*, *PpyKIN*, or *PpyRD29A* (Table S3), but we detected their strong in vivo and in vitro interactions with *PpyCBFs*. By mutating the CGAC binding site in *PpyCOR15A*, we were able to determine that *PpyCBFs* can also bind to the TCGAC binding site (Figure 6). In our previous study, we found that *PpCBF2* can also bind to the CCGA binding site in the *PpCBF4* promoter [22], which indicates that CGA is the actual core of the CBF binding site in pears.

To investigate the underlying mechanism of transcriptional regulation of *PpyCBF* expression by abiotic stress and ABA treatments, we examined the promoter regions of all *PpyCBFs* (Table 1). We found that *PpyCBF* expressions during abiotic stress are regulated by CRT/DRE, GT-1-like box¸ ICE1-like, NAC, and I BOX TFs, whereas during ABA treatment, ABRE and G-box1 TFs are involved. A bZIP transcription factor specifically recognizes G-box1 in promoters of ABA-responsive genes [43]. The absence of G-box1 *cis* elements and the presence of ABRE *cis* elements in *PpyCBF3* and *PpyCBF5* indicates that these genes are only regulated by the ABI3/VP1 cascade. In contrast, clade II *PpyCBFs* are regulated by both b-ZIP and ABI3 TFs, which explains why the expressions of clade II CBFs during ABA stress were relatively higher than those of *PpyCBF3* and *PpyCBF5* (Figure 2a). NAC TFs in pears are highly abiotic-stress responsive [44]. ICE-1 encoding a MYC-like basic helix–loop–helix protein that binds to Myc recognition sequences [33] and transcriptional induction of *PpCBFs* by *PpICE1s* have already been observed in pears [22]. *DREB1* genes are also negatively regulated by MYB15, an R2R3-type MYB transcription factor in *Arabidopsis* [7]. In both *Arabidopsis* and soybeans, a bZip TF recognizes GT-1-like boxes and plays a role in salt- and pathogen-induced gene expression [45]. MIKC *cis* elements in *PpyCBFs* also display a dormancy response, as the CBF–DAM regulon aids pear adaptation through bud endodormancy [22]. Given the above mentioned results, the relatively high abundance of *PpyCBFs* in the face of abiotic stress as well as exogenous and endogenous ABA, the induction of ABA-dependent and -independent genes in overexpressed *Arabidopsis* under control conditions, and the in vivo and in vitro interactions of PpyCBFs with PpyCORs and the presence of both stress- and ABA-related *cis* elements in their promoters. 

## 4. Materials and Methods

### 4.1. Identification and Characterization of PpyCBFs

Protein sequences of PpyCBF subfamily members and PpyCORs were retrieved from the Pear Genome Project database (http://peargenome.njau.edu.cn/), while two databases were used to obtain *Malus* (Md), *Prunus* (Ppe), *Fragaria* (Fv), and *Vitis* (Vv) CBFs: The Genome Database for Rosaceae (GDR; http://www.rosaceae.org/) and the Plant Transcription Factor database (Plant TFDB v4.0; http://planttfdb.cbi.pku.edu.cn/). AtCBFs were downloaded from the Arabidopsis Information Resource (https://www.arabidopsis.org/). Collinear blocks of PpyCBFs and whole genomes within species were identified in MCScanX with default settings and an *E*-value ≤ 1 × 10^−10^. After aligning all sequences in ClustalX, the resulting identity matrix was checked using BioEdit software. Phylogenetic analysis of PpyCBFs and CBFs of other crop species was performed by the neighbor-joining method with 1000 bootstrap replicates in MEGA v7.0. Gene structure and motif analyses were carried out using Gene Structure Display Server v2.0 (http://gsds.cbi.pku.edu.cn/) and MEME v5.0.4 (http://meme-suite.org/tools/meme) tools with default parameters. The PlantPan2.0 (http://plantpan2.itps.ncku.edu.tw/) database with 2000 nucleotides was used for promoter analysis.

### 4.2. Plant Materials and Abiotic Stress Treatments

For abiotic stress experiments, vegetative buds of Asian pear cultivar ‘Dangshan Suli’ were collected before bud break in March 2018. After collection, buds were washed, sterilized, and then grown in half-strength MS medium to generate pear seedlings. Seedlings of a uniform size with six to eight leaves were randomly selected for abiotic stress treatments. For the low temperature treatment, seedlings in MS medium were exposed to 4 °C, while drought and salt stress treatments were carried out by respectively adding 200 mM NaCl and 15% PEG6000to half-strength MS medium. Samples were collected with three replicates after 0, 6, 12, 24, and 48 h of treatment. For ABA stress treatments, wild-type pear calli were placed in half-strength MS medium containing 100 µM ABA (stressed) or 100 µM absolute ethanol (Mock), and sampling was carried out with three replicates of each treatment group after 0, 3, 6, 12, 24, and 48 h. Following the abiotic stress treatments, each sample was immediately frozen in liquid nitrogen and stored at −80 °C. Plant materials and methods for study of bud endodormancy in pears were the same as those of a previously published study [44].

### 4.3. Analysis of Stress Tolerance of Transgenic Plants

After amplification, *PpyCBF2* and *PpyCBF3* coding sequences were cloned into a pCAMBIA 1301 vector to generate 35S::PpyCBFs constructs. The recombinant plasmids were inserted into Agrobacterium EHA105 cells and then transformed into flowering *Arabidopsis thaliana* plants by the floral dip method. After 7 days, the floral dip procedure was repeated. Following seed collection, the transgenic *Arabidopsis* plants were screened on MS medium containing 1 μg mL^−1^ of the antibiotic hygromycin. Putative transformants among the T_1_ progeny, confirmed by RT-PCR using PpyCBF2- and PpyCBF3-ORF-F/R primers, were regrown using the same procedure to obtain T_3_ progeny. The line of T_3_ plants with the highest PpyCBF2 and PpyCBF3 abundances was selected and grown to generate T_4_ progeny, which were used to assess in vivo abiotic stress tolerance. For this assessment, seeds of wild-type and overexpressed lines were germinated on MS medium for 14 days, and their seedlings were then grown for 5 days on vertical plates containing MS medium supplemented with either 50 mM NaCl (to assess salt tolerance) or 10% PEG (to assess drought tolerance). As a control, another set of seedlings were grown on MS medium with no supplement. To assess cold tolerance, seedlings on MS plates were exposed to 10 °C for 21 days. After abiotic stress treatments, all seedlings were grown under normal conditions on MS medium for 5 days to check their recovery rate. ImageJ v1.8.0 software was used to measure root lengths of wild-type and overexpressed lines under normal and abiotic stress conditions.

### 4.4. Histochemical Analysis of H_2_O_2_ and O_2_^•−^

For histochemical analysis of H_2_O_2_ and O2^•−^, fresh diaminobenzidine (DAB) and nitroblue tetrazolium (NBT) solutions were prepared following a method reported previously [46]. Plant leaves were immersed in DAB and NBT solutions and incubated overnight at room temperature in darkness, the latter achieved by wrapping in aluminum foil. To remove chlorophyll for proper visualization, the leaves were bleached in absolute ethanol for 10 min at 95 °C in a water bath. Photographs of stained samples were taken using a Leica DMLB fluorescence microscope, where brown and blue spots respectively indicated the presence of H_2_O_2_ and O_2_^•−^ in situ.

### 4.5. RNA Extraction and cDNA Synthesis

Total RNA was extracted from three biological replicates using a modified cetyltrimethylammonium bromide method as described in our previous study [47]. cDNA was then synthesized from 4 μg of DNA-free RNA using an iScript cDNA Synthesis kit (Bio-Rad, Foster, CA, USA) following the manufacturer’s instructions. Ten-fold diluted cDNA was used as a template for qRT-PCR analysis.

### 4.6. qRT-PCR Analysis

qRT-PCR amplifications were performed in 15 μL reaction volumes composed of 7.5 μL SYBR Premix Ex *Taq* (TliRNaseH Plus, Takara Biotechnology (Dalian) Co., Ltd. Dalian, China), 1 μL cDNA, 0.5 μL each of forward and reverse primers, and 5.5 μL RNase-free water. The amplifications were carried out on a CFX Connect real-time PCR system (Bio-Rad, Hercules, CA, USA) according to the following protocol: 95 °C for 30 s, followed by 40 cycles of 95 °C for 5 s and 60 °C for 20 s. Melting curves were used to confirm the specificity of the qRT-PCR primers. Relative gene transcript levels were determined using the 2^−ΔΔCt^ method and normalized against *PpyActin* (JN684184).

### 4.7. Site-Directed Mutagenesis of Gene Promoters

To check possible binding sites of PpyCBFs in *PpyCOR* promoters, the predicted sites were altered by directed mutagenesis. Motif mutations were carried out using a mutagenesis system after designing specific primers for possible binding sites. Transactivation effects of PpyCBFs on mutated promoters were further examined using dual luciferase and Y1H assays.

### 4.8. Transient Expression and Luciferase Measurement

A dual luciferase assay was used to detect in vivo transactivation effects of transcription factors. Full-length *PpyCBF* and *PpyCOR* promoters (2000 nucleotides) were inserted into pGreenII 0029 62-SK and pGreenII 0800-LUC vectors, respectively. The dual luciferase assay was carried out with *Nicotiana benthamiana* leaves according to our previously described protocol [22]. Three independent experiments with a minimum of four replicates were performed to verify the results.

### 4.9. Yeast One-Hybrid Assay

Y1H assays were conducted using a Matchmaker Gold Yeast One-Hybrid System kit (Clontech, Takara, Japan) according to the instructions in the user manual. Subsequent analyses were completed as previously described [48].

### 4.10. Statistical Analysis

Experiments were set up according to a completely randomized design. Analysis of variance followed by Duncan’s multiple range test was used to test the overall significance of differences among treatments (*p* < 0.05). Significant differences between treatments were assessed by Student’s *t*-test at *p* < 0.05, *p* < 0.01, and *p* < 0.001. All data were analyzed in SPSS v25 (SPSS Inc., Chicago, IL, USA).

## 5. Conclusions

We identified six *PpyCBF* homologues (*PpCBF1*-*6*) encoding potential transcription factors in Asian pear. All *PpyCBF* members accentuated during different abiotic stresses and endo and exogenous ABA. II clade *PpyCBFs* were not only more low temperature (LT) and ABA responsive but also enhanced LT stress tolerance in overexpressed Arabidopsis as compared to I and III clades *PpyCBFs.* Ectopic expressions of *PpyCBF2* and *PpyCBF3* in Arabidopsis also increased the expressions of endogenous ABA dependent and independent genes during normal conditions. A conversed CBF-COR regulatory cascade was also observed in pear. We conclude that *PpyCBFs* may follow both ABA-dependent and -independent stress signaling pathways during abiotic stress in pears. PpyCBF transcription factors may thus act redundantly during abiotic stress through ABA-dependent and -independent pathways. The results of our investigation, the first to differentiate the functions of the complete CBF subfamily in any rosaceous crop species, should have an important influence on the study of stress in woody species and may be applicable for the genetic engineering of different functions of transcription factors in other plant species.

## Figures and Tables

**Table 1:** Promoter analysis of all isolated *PpyCBFs*.

TFs family	Functions	*cis*-Element	Sequences	*PpyCBF3*	*PpyCBF1*	*PpyCBF2*	*PpyCBF4*	*PpyCBF5*	*PpyCBF6*
**ABI3/VP1**	ABA responsive	ABRE	CATGC	1	4	1	4	1	1
**AP2/EREBP**	Cold, drought, NaCl	CRT/DRE	CCGAC	6	4	1	4	8	3
**AP2/RAV**	Photoperiodism, flowering	B3	CAACA	10	8	5	7	9	8
**ARF**	Auxin response	SURE	GAGACA	3	2	2	2	2	1
**bHLH**	Iron toxicity	IRO2	CACGTGG	0	0	2	2	0	2
**bZIP**	ABA, NaCl, drought, heat	G-box1	CACGTG	0	1	2	2	0	3
**bZIP**	Salt, Pathogen	GT-1-like box	GAAAAA	3	3	7	3	4	4
**ERF**	Defense responses	GCC box	AGCCG	7	1	0	4	9	0
**GATA**	Light response	GATA box	GATA	14	16	16	11	12	15
**MADS**	Plant development	MIKC	CC[A/T]5	1	0	1	3	1	2
**MYB like**	Light response	I BOX	AAACCA	1	0	2	1	0	0
**MYB/SANT**	Gibberellin response	GARC	AACAAA	6	3	6	4	2	3
**MYC-like bHLH**	Cold stress	ICE1-like	CATTTG	1	1	4	1	2	1
**NAC**	Cold, drought, NaCl	NAC	CATGT	2	3	3	2	3	3
**TCP/PCF1**	Oxidative stress	Site 2	TGGGC	3	1	3	1	1	2
**WRKY**	Bacterial blight	PRE2	ACGCTG	1	0	0	0	2	0
**WRKY**	Bacterial blight	PRE4	TGCGCT	1	0	0	0	2	1

## Supplementary Materials

Supplementary materials can be found at https://www.mdpi.com/1422-0067/20/9/2074/s1.

## Abbreviations

CORs	Cold Regulons
HMM	Hidden Markov Model
MEGA	Molecular Evolutionary Genetics Analysis
TF	Transcription factor
Y1H	Yeast one hybrid
SnRK2	Snf1-Related kinase 2
CTAB	Cetyltrimethyl Ammonium Bromide
