# Supplementary material for: Phylogenetic, Molecular, and Functional Characterization of PpyCBF Proteins in Asian Pears (Pyrus pyrifolia)
Source: Int J Mol Sci. 2019 Apr 26;20(9):2074. doi: 10.3390/ijms20092074 (PMC6539064; doi:10.3390/ijms20092074)
Supplement: Supplementary file 1 [file ijms-20-02074-s001.pdf]

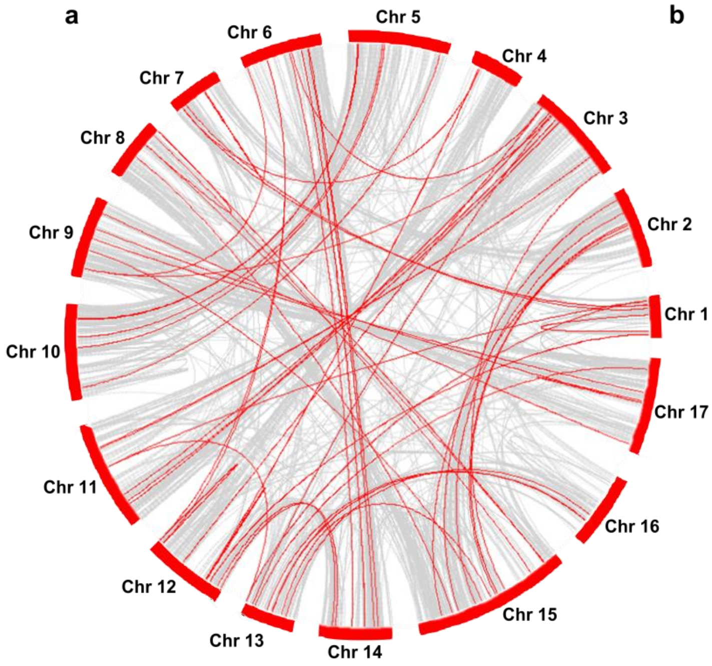

b

1	*Pbr039133.1:Pbr041863.1*	35	*Pbr015291.2:Pbr025174.1*
2	*Pbr014186.2:Pbr033937.1*	36	*Pbr003636.1:Pbr004690.1*
3	*Pbr008289.1:Pbr033937.1*	37	*Pbr001083.1:Pbr019788.1*
4	*Pbr000615.1:Pbr023212.1*	38	*Pbr019494.1:Pbr030451.1*
5	*Pbr025136.1:Pbr030418.1*	39	*Pbr000436.1:Pbr016184.1*
6	*Pbr000435.1:Pbr016185.1*	40	*Pbr017391.1:Pbr030208.1*
7	*Pbr035774.1:Pbr035788.1*	41	*Pbr023902.1:Pbr035788.1*
8	*Pbr013149.1:Pbr017297.1*	42	*Pbr018624.1:Pbr037828.1*
9	*Pbr013631.1:Pbr031557.1*	43	*Pbr010809.1:Pbr031557.1*
10	*Pbr025993.1:Pbr026023.1*	44	*Pbr004315.1:Pbr015044.1*
11	*Pbr004315.1:Pbr017146.1*	45	*Pbr024467.1:Pbr039770.1*
12	*Pbr002083.1:Pbr039770.1*	46	*Pbr018333.1:Pbr037846.1*
13	*Pbr018641.1:Pbr037846.1*	47	*Pbr010138.1:Pbr029841.1*
14	*Pbr010809.1:Pbr013631.1*	48	*Pbr015044.1:Pbr017146.1*
15	*Pbr020595.1:Pbr027839.1*	49	*Pbr001362.1:Pbr015698.1*
16	*Pbr029055.1:Pbr033430.1*	50	*Pbr033396.1:Pbr033431.1*
17	*Pbr011102.1:Pbr025354.1*	51	*Pbr000753.1:Pbr030160.1*
18	*Pbr035775.1:Pbr035787.1*	52	*Pbr023899.1:Pbr035787.1*
19	*Pbr000396.1:Pbr016224.1*	53	*Pbr011224.1:Pbr014831.1*
20	*Pbr001363.1:Pbr015697.1*	54	*Pbr026000.1:Pbr026017.1*
21	*Pbr024078.1:Pbr031830.1*	55	*Pbr011145.1:Pbr016049.2*
22	*Pbr007146.1:Pbr020227.1*	56	*Pbr001361.1:Pbr015699.1*
23	*Pbr010139.1:Pbr029833.1*	57	*Pbr007699.1:Pbr016912.1*
24	*Pbr014125.1:Pbr026719.1*	58	*Pbr032608.1:Pbr034761.1*
25	*Pbr022083.1:Pbr023949.1*	59	*Pbr009571.1:Pbr010949.1*
26	*Pbr002460.1:Pbr019145.1*	60	*Pbr013924.1:Pbr021781.1*
27	*Pbr013924.1:Pbr032764.1*	61	*Pbr025944.1:Pbr031258.1*
28	*Pbr013255.1:Pbr030636.1*	62	*Pbr013289.1:Pbr028836.1*
29	*Pbr005504.1:Pbr016795.1*	63	*Pbr015293.1:Pbr025176.1*
30	*Pbr025990.1:Pbr026026.1*	64	*Pbr027726.1:Pbr037414.1*
31	*Pbr013714.1:Pbr030636.1*	65	*Pbr013927.1:Pbr032762.1*
32	*Pbr020278.1:Pbr038937.1*	66	*Pbr007346.1:Pbr034260.1*
33	*Pbr025940.1:Pbr031253.1*	67	*Pbr004208.1:Pbr031253.1*
34	*Pbr004208.1:Pbr025940.1*	68	*Pbr025458.1:Pbr033071.1*

Figure S1. Collinerity analysis of *PpyAP2/ERF* TFs. **a** Red lines indicate the *PpyERFS* duplicated genes while the gray lines show the whole genome duplication events. Red bars indicate each chromosome number. **b** Duplicated gene pairs of *PpERFs* TFs while the red highlighted duplicated gene pairs belong to *PpyCBFs* subfamily.

Table S1. Sequence identity Matrix of all isolated *PpyCBF* TFs:

Seq->	*Pbr013924*	*Pbr032764*	*Pbr003714*	*Pbr003734*	*Pbr030749*	*Pbr021780*	*Pbr037203*	*Pbr037204*	*Pbr037205*	*Pbr021781*	*Pbr037202*	*Pbr030748*	*Pbr003716*	*Pbr003732*	*Pbr016792*
**Given name**	*PpyCBF3*	*PpyCBF5*	*PpyCBF4*	*PpyCBF15*	*PpyCBF12*	*PpyCBF9*	*PpyCBF10*	*PpyCBF11*	*PpyCBF8*	*PpyCBF1*	*PpyCBF7*	*PpyCBF2*	*PpyCBF13*	*PpyCBF14*	*PpyCBF6*
Chr. Pos.	**Chr7**	**Chr1**	**Scaf. 117.0.1**	**Scaf. 117.0.1**	**Chr6**	**Chr4**	**Scaf.752.0**	**Scaf.752.0**	**Scaf.752.0**	**Chr4**	**Scaf.752.0**	**Chr6**	**Scaf. 117.0.1**	**Scaf. 117.0.1**	**Chr14**
PpyCBF3	1	0.799	0.557	0.556	0.396	0.365	0.365	0.407	0.49	0.529	0.498	0.558	0.557	0.557	0.458
PpyCBF5	---	1	0.559	0.557	0.387	0.354	0.354	0.393	0.487	0.525	0.495	0.563	0.563	0.563	0.447
PpyCBF4	---	---	1	0.994	0.621	0.573	0.573	0.618	0.774	0.837	0.79	0.936	0.942	0.942	0.475
PpyCBF15	---	---	---	1	0.616	0.57	0.57	0.615	0.77	0.832	0.785	0.931	0.936	0.936	0.471
PpyCBF12	---	---	---	---	1	0.271	0.271	0.316	0.492	0.534	0.503	0.597	0.594	0.594	0.297
PpyCBF9	---	---	---	---	Incomplete seq	1	1	0.921	0.647	0.701	0.66	0.579	0.579	0.579	0.363
PpyCBF10	---	---	---	---	---	---	1	0.921	0.647	0.701	0.66	0.579	0.579	0.579	0.363
PpyCBF11	---	---	---	---	---	---	---	1	0.689	0.747	0.704	0.624	0.624	0.624	0.393
PpyCBF8	---	---	---	---	---	---	---	---	1	0.922	0.949	0.772	0.772	0.772	0.426
PpyCBF1	---	---	---	---	---	---	---	---	---	1	0.941	0.834	0.834	0.834	0.454
PpyCBF7	---	---	---	---	---	---	---	---	---	---	1	0.788	0.788	0.788	0.436
PpyCBF2	---	---	---	---	---	Same Clade						1	0.984	0.984	0.47
PpyCBF13	---	---	---	---	---	---	---	---	---	---	---	---	1	1	0.469
PpyCBF14	---	---	---	---	---	---	---	---	---	---	---	---	---	1	0.469
PpyCBF6	---	---	---	---	---	---	---	---	---	---	---	---	---	---	1

Table S2. Total number of stress related *cis*-elements in promoters of *PpyCBF* TFs:

TFs	Cold	Salt	Drought	Oxidative	Light	Iron toxicity	Pathogen	Heat	ABA	GA	Auxin
PpyCBF3	9	11	8	3	15	1	5	0	1	6	3
PpyCBF1	8	11	8	1	16	4	3	1	5	3	2
PpyCBF2	8	13	6	3	18	1	7	2	7	6	2
PpyCBF4	6	11	8	1	12	4	3	2	7	4	2
PpyCBF5	13	15	11	1	12	1	8	0	1	2	2
PpyCBF6	7	13	9	2	15	1	5	3	7	3	1

Table S3. Promoter analysis of *PpyCORs:*

TFs family	Functions	*cis*-element	Sequences	*PpyCOR47*	*PpyCOR15A*	*PpyRD29A*	*PpyKIN*
**ABF/AREB**	ABA responsive	ABRE	CATGC	5	5	3	4
**AP2/EREBP**	Cold, drought, NaCl	CRT/DRE	CCGAC	6	0	0	0
			CGAC	10	6	7	3
**AP2/RAV**	Photoperiodism	B3	CAACA	2	8	6	6
**bHLH**	Iron toxicity	IRO2	CACGTG	3	3	0	2
**bZIP**	ABA, NaCl, drought,	G-box1	CACGTG	3	3	0	2
**bZIP**	Salt, Pathogen	GT-1-like box	GAAAAA	2	3	4	8
**ERF**	Defense responses	GCC box	AGCCG	1	2	3	0
**GATA**	Light response	GATA box	GATA	10	17	15	14
**MADS**	Plant development	MIKC	CC[A/T]5	1	1	2	1
**MYB like**	Light response	I BOX	AAACCA	3	2	2	4
**MYB/SANT**	Gibberellin response	GARC	AACAAA	2	2	6	0
**MYC-like bHLH**	Cold stress	ICE1-like	CATTTG	4	3	1	0
**NAC**	Cold, drought,NaCl,	NAC	CATGT	3	5	3	8
**TCP/PCF1**	Oxidative stress	Site 2	TGGGC	6	5	2	2
**WRKY**	Bacterial blight	PRE2	ACGCTG	2	1	0	1
		PRE4	TGCGCT	1	0	2	0

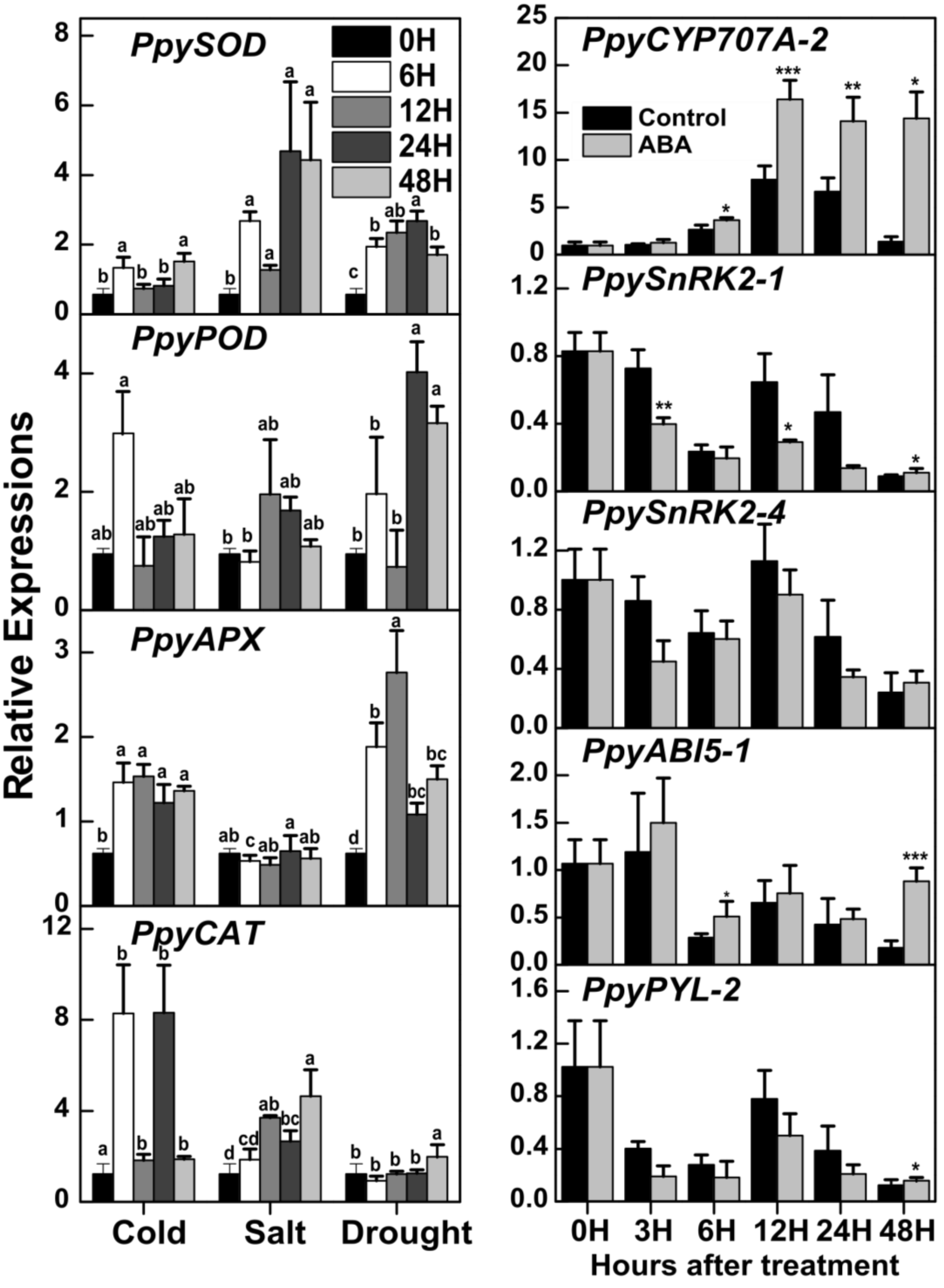

Figure S2. **Expression analysis of antioxidant and ABA-responsive genes in pear explants and pear calli during abiotic stresses and ABA treatment.** Both relative expressions were normalized to *PpyActin* expression level. Error bars indicate standard errors from three biological replicates (*$P < 0.05$, **$P < 0.01$, ***$P < 0.001$) while means with different letters had significant differences ($P < 0.05$).

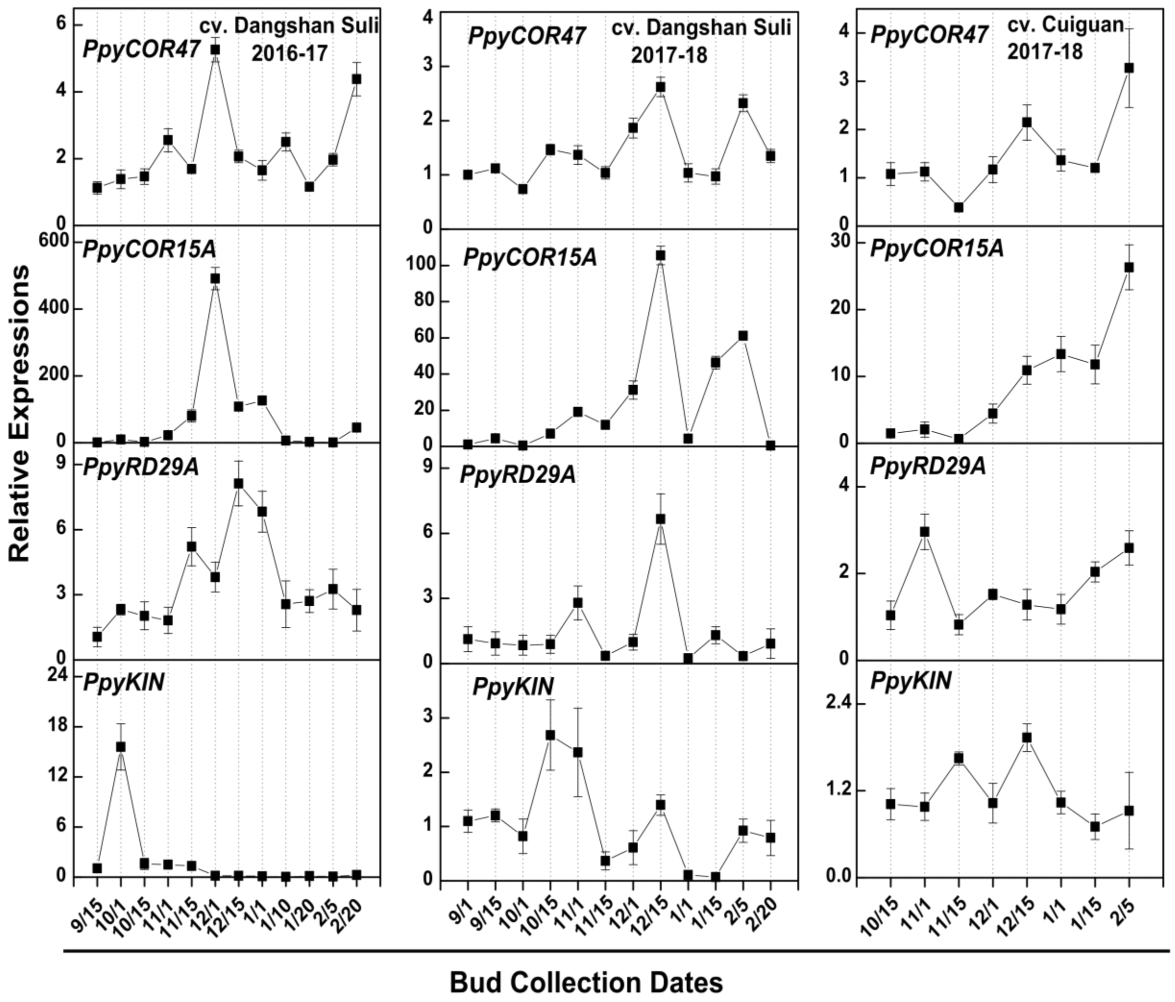

Figure S3. Relative expressions of *PpyCORs* during bud endodormancy in *Pyrus pyrifolia* cv 'Dangshan Suli' and 'Cuiguan' during two successive years 2016-2017 and 2017-2018. Buds were collected from 9/15 to 2/25 with about 15 days interval. The data were normalized to *PpyActin* expression level and the mean expression value was premeditated from 4 independent replicates. The standard deviation was shown by vertical bars.

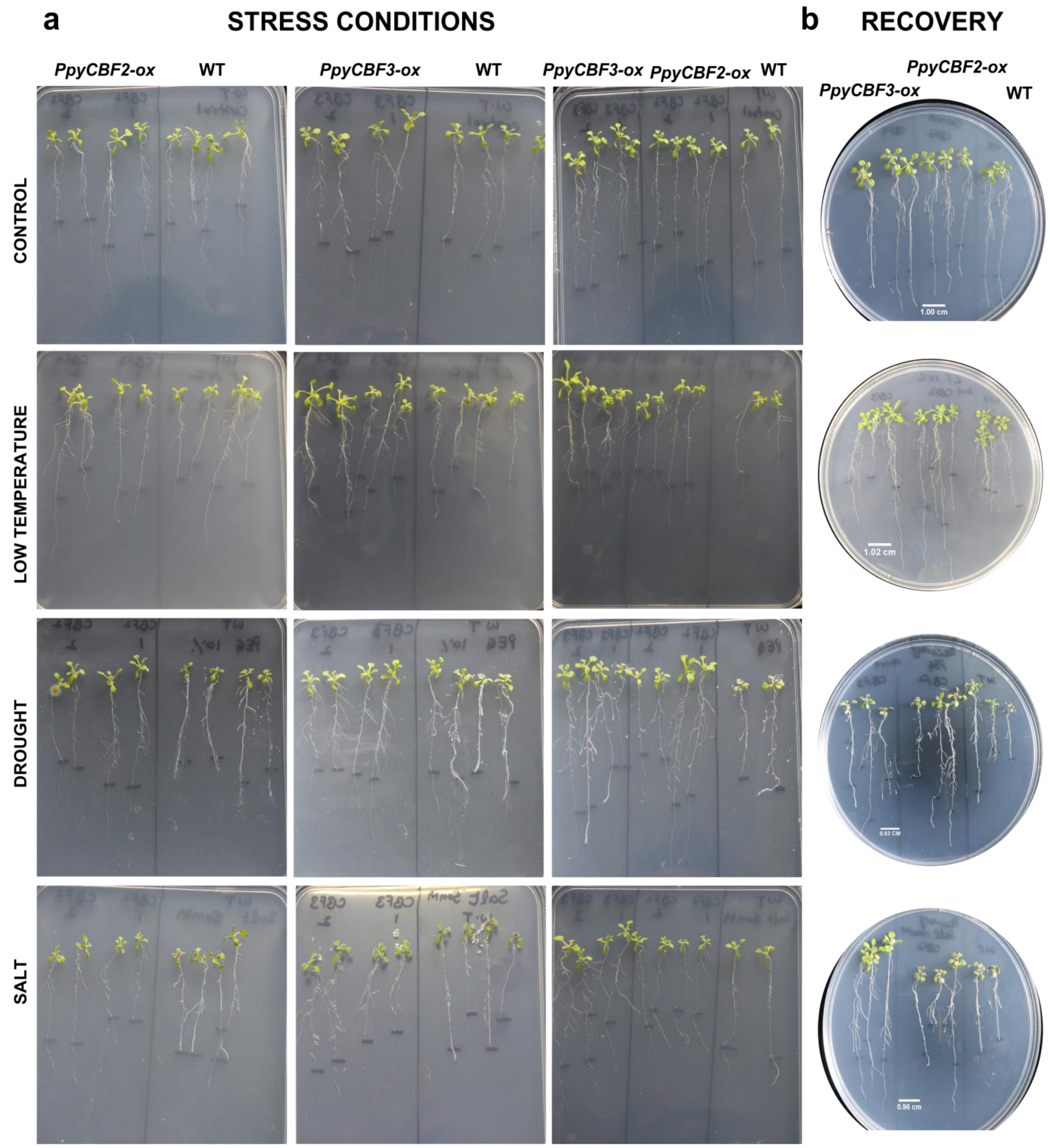

Figure S4. Phenotypic analysis of *PpyCBF2 and PpyCBF3* overexpressing arabidopsis and WTs during abiotic stresses and recovery periods. **a** Phenotype of overexpressed and WTs arabidopsis during control, LT, drought and salt stresses. Black lines indicate the root lengths before stresses while below these lines are increase in root lengths during abiotic stress conditions. **b**. After abiotic stress, explants were transformed into normal conditions without abiotic stress conditions to check their recovery.

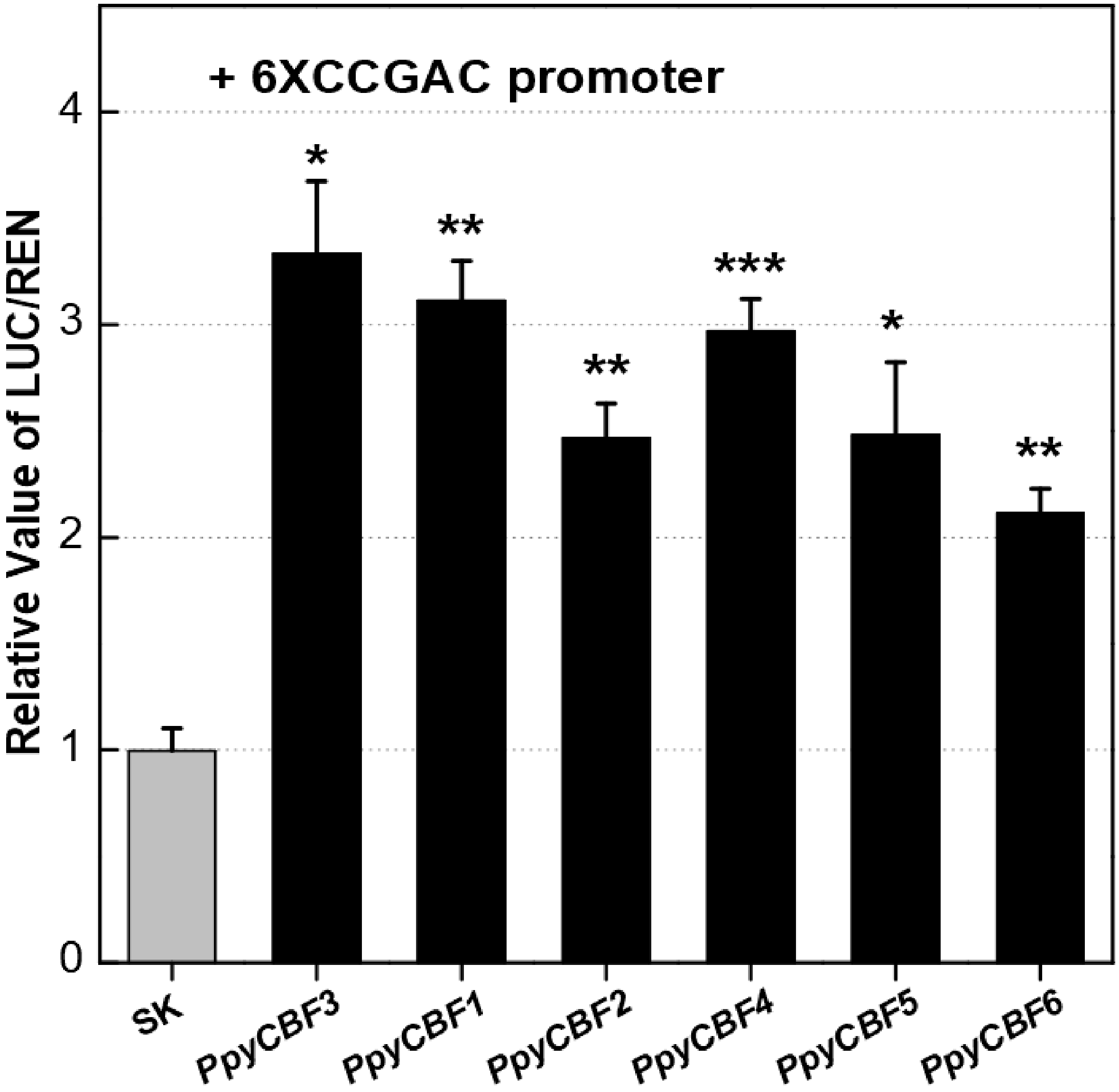

Figure S5. Transcriptional activity of *PpyCBFs* with 6XCRT binding sites. The ratio of LUC/REN of the empty vector (SK) plus promoter was used as calibrator (set as 1). Three independent experiments were done to verify the results. Error bars showed SEs with at least four biological replicates while asterisks showed significant differences of genes SK with empty SK (*$P < 0.05$, **$P < 0.01$, ***$P < 0.001$).
